# Nutritional Status as a Risk Factor for the Early Discontinuation of Inhaled Liposomal Amikacin in Mycobacterium avium Complex Pulmonary Disease

**DOI:** 10.7759/cureus.91797

**Published:** 2025-09-07

**Authors:** Hideaki Yamakawa, Chisa Uzuka, Daisuke Nakatani, Moe Nomaki, Tomohiro Oba, Rie Kawabe, Shintaro Sato, Keiichi Akasaka, Masako Amano, Jun Araya, Hidekazu Matsushima

**Affiliations:** 1 Department of Respiratory Medicine, Saitama Red Cross Hospital, Saitama, JPN; 2 Division of Respiratory Diseases, Department of Internal Medicine, Jikei University School of Medicine, Tokyo, JPN

**Keywords:** amikacin liposome inhalation suspension, mycobacterium avium complex pulmonary disease, nutrition, therapy, tolerability

## Abstract

Background: Pulmonary nontuberculous mycobacterial (NTM) disease, especially *Mycobacterium avium* complex pulmonary disease (MAC-PD), remains difficult to treat, with limited success and frequent adverse events. Amikacin liposome inhalation suspension (ALIS) is commonly used for refractory MAC-PD; however, early discontinuation in routine care is not uncommon. This study aimed to identify baseline clinical and nutritional factors associated with early ALIS discontinuation in real-world settings.

Methods: We retrospectively analyzed patients with MAC-PD who received ALIS at Saitama Red Cross Hospital (November 2021-December 2024). Patients were categorized into early discontinuation (<6 months) and continuation (≥6 months) groups. Baseline data included age, sex, body mass index (BMI), albumin, comorbidities, pulmonary function, radiographic patterns, and co-infection status. Statistical tests included Welch's t-test, the Mann-Whitney U test, and Fisher's exact test.

Results: Among 25 patients, 24% discontinued ALIS within six months. The early discontinuation group had significantly lower BMI (14.9±1.8 kg/m² vs. 19.0±3.3; p=0.002) and albumin (3.53±0.54 g/dL vs. 4.04±0.39; p=0.001). Fibrocavitary (FC) patterns were more frequent (50% vs. 11%;* *p=0.048). Adverse events were more common (p=0.042), with hemoptysis prominent. Other reasons included hoarseness, cough, fatigue, fever, dyspnea, ALIS-related hypersensitivity pneumonitis, and gastrointestinal symptoms.

Conclusions: Low BMI, hypoalbuminemia, and FC radiographic patterns were significant risk factors for early ALIS discontinuation. These findings emphasize that maintaining adequate nutritional status is a prerequisite for successful multidrug antimicrobial therapy for pulmonary NTM disease. Clinicians should carefully evaluate these factors before starting ALIS and ensure close monitoring and nutritional support to improve adherence and outcomes.

## Introduction

Pulmonary nontuberculous mycobacterial (NTM) disease remains a major therapeutic challenge, requiring prolonged multidrug regimens with variable efficacy and often adverse events. Current American Thoracic Society (ATS)/European Respiratory Society (ERS)/European Society of Clinical Microbiology and Infectious Diseases (ESCMID)/Infectious Disease Society of America (IDSA) guidelines recommend three-drug macrolide-based therapy for macrolide-susceptible *Mycobacterium avium* complex pulmonary disease (MAC-PD) [1,2], but success rates remain suboptimal, with culture conversion in only about 61% of cases [3]. For refractory MAC-PD failing to convert after ≥6 months of guideline-based therapy (GBT), amikacin liposome inhalation suspension (ALIS) is recommended to improve culture conversion by delivering high concentrations of amikacin to the lungs while minimizing systemic toxicity [1]. The CONVERT trial showed that ALIS improved culture conversion rates at six months in patients with MAC-PD, but 34.6% of patients discontinued treatment before completing the protocol-defined period, highlighting tolerability challenges that may be even greater in routine practice [4,5].

Identifying clinical factors associated with early ALIS discontinuation is crucial to improve patient selection and management. Although several prognostic factors have been identified in NTM lung disease such as age, sex, nutritional status (including low body mass index (BMI) and hypoalbuminemia), muscle mass, lung function, comorbidities, *Aspergillus *and *Pseudomonas aeruginosa* co-infection, characteristic radiological features, and inflammatory markers, nutritional status and body composition are particularly recognized as important prognostic factors, with low BMI and hypoalbuminemia linked to worse outcomes [6,7]. However, whether these factors also predict tolerability and persistence with ALIS remains unclear. This study aimed to evaluate baseline clinical and nutritional factors associated with early (<6 months) ALIS discontinuation in a real-world cohort, comparing patients who continued ALIS for ≥6 months versus those who discontinued earlier to identify risk factors to guide treatment decisions.

## Materials and methods

Study sample

We conducted a retrospective observational study of patients with MAC-PD who received ALIS at Saitama Red Cross Hospital between November 2021 and December 2024. Patients were included if they met the diagnostic criteria for NTM pulmonary disease according to ATS/ERS/ESCMID/IDSA guidelines and received ALIS as part of routine clinical care [1]. According to these guidelines, diagnostic confirmation required either at least two separate sputum cultures positive for NTM or one positive bronchoscopic culture, in combination with compatible clinical and radiological findings. ALIS was used for patients with refractory MAC-PD, defined as those who failed to achieve negative culture conversion after combination therapy. The Institutional Review Board of Saitama Red Cross Hospital approved this study (approval number: 25-Q; date: July 18, 2025). Informed consent was waived by the Ethics Committee of Medical Research because of the retrospective nature of this study.

Definition of early discontinuation of ALIS administration

Patients were categorized into two groups based on treatment duration: early discontinuation (<6 months of ALIS therapy) and continuation (≥6 months). Baseline clinical characteristics, including age, sex, BMI, albumin levels, comorbidities, pulmonary function, radiological findings, and co-infection status, were extracted from medical records. Nutritional status and body composition markers were specifically examined for their association with early discontinuation. As ALIS administration, a 590 mg dose of ALIS (ARIKAYCE, Insmed Inc., Bridgewater, New Jersey, United States) was inhaled once daily using the LAMIRA nebulizer system (PARI, Midlothian, Virginia, United States) according to the manufacturer's instructions. When daily administration was not feasible owing to adverse events, alternate-day or thrice-weekly administration was permitted as an alternative schedule. In addition, the Geriatric Nutritional Risk Index (GNRI) score was calculated as follows: $\text{GNRI}=\left( 1.489\times\text{serum albumin (g/L)} \right)+41.7\times\left( \frac{\text{present weight}}{\text{ideal body weight}} \right)$ [8].

Microbiological examinations

Sputum specimens were routinely cultured for mycobacteria and, when available, for other bacteria and fungi. Minimum inhibitory concentration (MIC) determination was carried out by the broth microdilution method following the Clinical and Laboratory Standards Institute (CLSI) Standard M24 guidelines, which is the standard reference method for nontuberculous mycobacteria [9]. Clarithromycin (CAM) resistance was defined as MIC ≥32μg/mL, and amikacin resistance was defined as MIC ≥128μg/mL, which is the CLSI recommended breakpoint [9-11]. Sputum culture conversion within one year was considered achieved with three consecutive negative sputum cultures of MAC [12]. In addition, co-infection was defined as the detection of pathogenic microorganisms other than MAC in at least two sputum cultures 6-24 months after the initiation of treatment for MAC-PD [12,13]. Radiographic patterns of NTM lung disease were classified as nodular/bronchiectatic (NB) type, fibrocavitary (FC) disease, and NB+FC according to the previous reports [6,14].

Statistical analysis

Group comparisons were performed using Welch's t-test for continuous variables with approximately normal distributions, the Mann-Whitney U test for non-normally distributed continuous variables, and Fisher's exact test for categorical variables. A two-sided p-value of <0.05 was considered statistically significant. All statistical analyses and data visualizations were conducted with Python (pandas, scipy, and matplotlib libraries).

## Results

Patient characteristics

A total of 25 patients with MAC-PD were included in this study. All patients had persistent positive sputum cultures for MAC prior to ALIS initiation. All patients who received ALIS during the study period were included; no patients were excluded. There were no missing data for the key variables (BMI, albumin, C-reactive protein (CRP), GNRI). The mean duration of pulmonary MAC disease at ALIS introduction was 6.18 years, with a standard deviation (SD) of 6.10 years. The distribution of MAC-PD at ALIS introduction was as follows: two patients (8%) for less than one year, six patients (24%) for one to less than three years, five patients (20%) for three to less than five years, three patients (12%) for five to less than seven years, another three patients (12%) for seven to less than nine years, and finally six patients (24%) for nine years or more. Of these, 19 patients (76%) continued ALIS treatment for ≥6 months, while six patients (24%) discontinued ALIS within six months. The overall mean age was 72±6.4 years, with no significant difference between the continuation and early discontinuation groups (71.4±6.7 vs. 74±4.9 years; p=0.320). Coexisting lung disease was present in 20% of patients overall, including chronic pulmonary aspergillosis (8%), interstitial lung disease associated with systemic sclerosis (8%), and primary ciliary dyskinesia (4%), with no significant group differences (p=0.560). As summarized in Table 1, patients in the early discontinuation group had significantly lower BMI (14.9±1.8 vs. 19.0±3.3 kg/m²; p=0.006; U=13.5), lower serum albumin levels (3.5±0.5 vs. 4.0±0.4 g/dL; p=0.047; U=25.5), and lower GNRI scores (56.6±8.5 vs. 65.3±6.0; p=0.039; U=24.0) compared with the continuation group. In contrast, CRP levels did not differ significantly between groups (3.1±2.7 vs. 1.0±1.1 mg/dL; p=0.138; U=81.0).

**Table 1:** Patient characteristics BMI: body mass index; GNRI: Geriatric Nutritional Risk Index; CRP: C-reactive protein; MAC-PD: *Mycobacterium avium* complex pulmonary disease; AZM: azithromycin; CAM: clarithromycin; RFP: rifampicin; EB: ethambutol; STFX: sitafloxacin; NB: nodular bronchiectatic; FC: fibrocavitary; ALIS: amikacin liposome inhalation suspension

Characteristics	Total (n=25)	ALIS ≥6 months (n=19)	ALIS <6 months (n=6)	P-value (U statistic)
Age (years), mean±SD	72±6.4	71.4±6.7	74±4.9	0.320
Female, n (%)	18 (72%)	13 (68%)	5 (83%)	1.0
Current or ex smoker, n (%)	8	6 (32%)	2 (33%)	1.0
Coexisting lung disease				
None	20 (80%)	16 (84%)	4 (67%)	0.560
Chronic pulmonary aspergillosis	2 (8%)	1 (5%)	1 (17%)	
Interstitial lung disease associated with systemic sclerosis	2 (8%)	1 (5%)	1 (17%)	
Primary ciliary dyskinesia	1 (4%)	1 (5%)	0	
Treatment with corticosteroids and/or immunosuppressive agents	1 (4%)	1 (5%)	0	1.0
BMI (kg/m^2^)	18.0±3.3	19.0±3.3	14.9±1.8	0.006 (U=13.5)
Albumin (g/dL)	3.9±0.5	4.0±0.4	3.5±0.5	0.047 (U=25.5)
GNRI	63.2±7.7	65.3±6.0	56.6±8.5	0.039 (U=24.0)
CRP (mg/dL)	1.5±1.8	1.0±1.1	3.1±2.7	0.138 (U=81.0)
Duration since diagnosis of MAC-PD	6.2±6.1	6.0±6.0	7.8±2.8	0.440
MAC species				
M. avium	18 (72%)	14 (74%)	4 (67%)	1.000
M. intracellulare	7 (28%)	5 (26%)	2 (33%)	
Clarithromycin resistance	7 (28%)	6 (32%)	1 (17%)	1.000
Baseline medications				
(AZM or CAM)+EB	5 (20%)	5 (26%)	0 (0%)	0.180
(AZM or CAM)+RFP+EB	9 (36%)	6 (32%)	3 (50%)	
(AZM or CAM)+RFP+STFX	3 (12%)	1 (5%)	2 (33%)	
(AZM or CAM)+RFP+EB+STFX	3 (12%)	2 (11%)	1 (17%)	
RFP+EB+STFX	4 (16%)	4 (21%)	0 (0%)	
AZM+RFP	1 (4%)	1 (5%)	0 (0%)	
Radiographic features				
NB	13 (52%)	12 (63%)	1 (17%)	0.048
NB+FC	7 (28%)	5 (26%)	2 (33%)	
FC	5 (20%)	2 (11%)	3 (50%)	
Number of abnormal lung fields (out of six lung zones)	3.9±1.4	4.0±1.4	3.8±1.3	0.72 (U=51.0)
Pathogens in mixed infections				
Aspergillus	5 (20%)	3 (16%)	2 (33%)	0.563
Pseudomonas aeruginosa	4 (16%)	4 (21%)	0 (0%)	0.541
ALIS treatment duration (during follow-up period)	Median 320 days (14-1184)	Median 353 days (216-1184)	Median 46 days (14-161)	<0.001
Everyday/alternate-day or thrice-weekly administration	19 (76%)/6 (24%)	14 (74%)/5 (26%)	5 (83%)/1 (17%)	1.000
Achieved culture conversion within 1 year	10 (40%)	10 (53%)	0 (0%)	0.051
Adverse events (including duplicates)				
None	7 (28%)	7 (37%)	0 (0%)	0.042
Hoarseness	10 (40%)	8 (42%)	2 (33%)	
Hemoptysis	4 (16%)	1 (5%)	3 (50%)	
Cough	5 (20%)	3 (16%)	2 (33%)	
Fever	2 (8%)	1 (5%)	1 (17%)	
Fatigue	2 (8%)	1 (5%)	1 (17%)	
ALIS-related hypersensitivity pneumonitis	1 (4%)	1 (5%)	0 (0%)	
Follow-up period	Median 422 days (240-1385)	Median 458 days (240-831)	Median 397 days (258-1385)	0.700
Number of deaths (during follow-up)	3 (12%)	1 (5%)	2 (33%)	0.178

**Figure 1:**
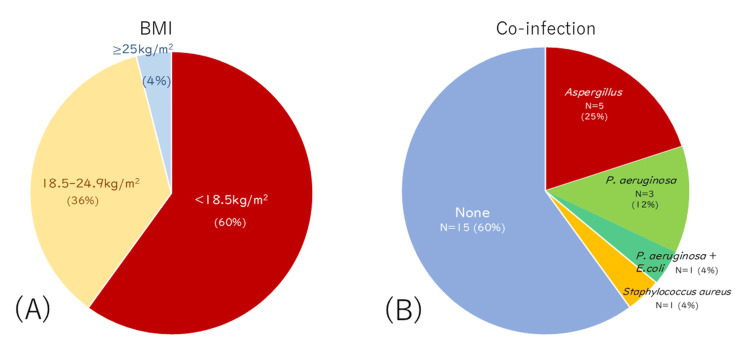
Baseline characteristics of the study cohort (A) Distribution of BMI at ALIS introduction. The majority of patients (60%) presented with a BMI of <18.5 kg/m², indicating underweight, 36% of patients had a normal BMI (18.5-24.9 kg/m²), and only 4% had a BMI of ≥25 kg/m². (B) Prevalence of co-infections at baseline. Co-infections were identified in 40% (N=10) of patients. The most common co-infecting pathogens included *Aspergillus* species (N=5; 25%) and *Pseudomonas aeruginosa* (N=4; 16%). Notably, when considering all instances of *P. aeruginosa*, including co-detection with *Escherichia coli*, a total of four patients (16%) were affected. Other detected pathogens included *P. aeruginosa* with *E. coli* (N=1; 4%) and *Staphylococcus aureus* (N=1; 4%). The remaining 60% (N=15) of patients had no co-infections. BMI: body mass index; ALIS: amikacin liposome inhalation suspension

Most patients (60%) had a BMI below 18.5 kg/m², indicating undernutrition. In contrast, 36% were in the normal range (18.5-24.9 kg/m²), and only 4% had a BMI ≥25 kg/m² (Figure 1A). As shown in Figure 1B, co-infections were identified in 40% of patients, most commonly *Aspergillus* species (25%) and *Pseudomonas aeruginosa* (12%).

Other detected pathogens included *P. aeruginosa* with *Escherichia coli *and *Staphylococcus aureus* (each 4%). Radiographic patterns showed a significant difference, with FC type being more frequent in the early discontinuation group (50% vs. 11%; p=0.048). No significant differences were observed in MAC species distribution or clarithromycin resistance.

The most common baseline regimen was macrolide (CAM or azithromycin: AZM)+RFP+ethambutol (EB) (36%), with other combinations including macrolide (CAM or AZM)+EB (20%) and regimens containing sitafloxacin (STFX) (total 40%). CAM was generally administered at 400 mg twice daily, although some patients received a reduced dose of 200 mg twice daily or had their dose decreased during treatment. AZM was administered at 250 mg once daily. RFP was usually given at 450-600 mg daily, but many patients received 300 mg daily or had dose reductions during the course of therapy. EB was administered at 15 mg/kg or, in many patients, at a fixed dose of 500 mg daily, reflecting routine clinical practice.

Median ALIS treatment duration was markedly shorter in the early discontinuation group (46 days; 14-161 days) compared to the continuation group (353 days; 216-1184 days; p<0.001). The proportion of daily vs. alternate-day administration was similar between groups (p= 1.000). Culture conversion within one year occurred in 10 patients (40%) overall, all within the continuation group (53% vs. 0%; p=0.051). Median observation periods were comparable between groups (458 days; 240-831 days vs. 397 days; 258-1385 days; p=0.700). Death during follow-up was not significantly different (5% vs. 33%; p=0.178).

Risk factors for the early discontinuation (<6 months) of ALIS

**Figure 2:**
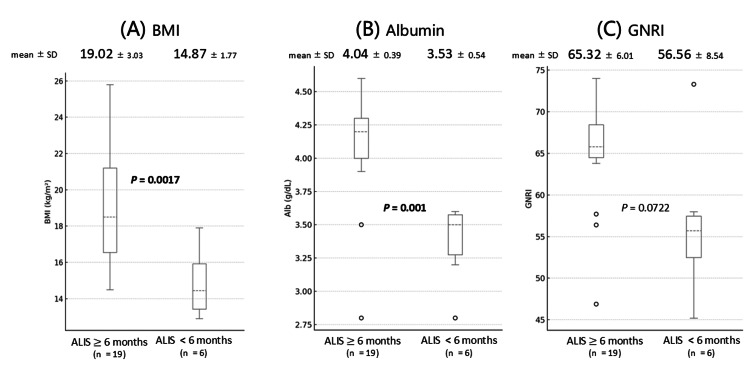
Comparison of nutritional and inflammatory markers between ALIS continuation and early discontinuation groups, analyzed using Welch's t-test Box plots representing the distribution of (A) BMI, (B) albumin levels, and (C) GNRI in patients who continued ALIS for ≥6 months (n=19) and those who discontinued ALIS within <6 months (n=6). The solid line within each box indicates the median, the dashed line indicates the mean, the box represents the IQR, and the whiskers extend to 1.5 times the IQR from the upper and lower quartiles. Outliers are shown as individual circles. (A) BMI: patients in the early discontinuation group showed significantly lower mean BMI (14.87±1.77 kg/m²) compared to the continuation group (19.02±3.03 kg/m²) (p=0.0017). (B) Albumin levels: the early discontinuation group exhibited significantly lower mean albumin levels (3.53±0.54 g/dL) compared to the continuation group (4.04±0.39 g/dL) (p=0.001). (C) GNRI: GNRI tended to be lower in the early discontinuation group (56.56±8.54) compared to the continuation group (65.32±6.01), although this difference did not reach statistical significance (p=0.0722). ALIS: amikacin liposome inhalation suspension; BMI: body mass index; GNRI: Geriatric Nutritional Risk Index; IQR: interquartile range

Analysis of nutritional and inflammatory markers revealed clear differences between groups. As shown in Figure 2, the early discontinuation group had significantly lower BMI (14.9±1.8 vs. 19.0±3.3 kg/m²; p=0.002) (Figure 2A) and albumin levels (3.53±0.54 vs. 4.04±0.39 g/dL; p=0.001) (Figure 2B). GNRI tended to be lower in the early discontinuation group (56.6± 8.5 vs. 65.3±6.0), although this difference did not reach statistical significance (p=0.072) (Figure 2C).

CRP levels were higher in the early discontinuation group (3.10±2.7 vs. 1.03±1.05 mg/dL), but this difference was not statistically significant (p=0.145). Radiographic assessment showed that FC or NB+FC type was more common in patients who discontinued early (p=0.048).

These differences in nutritional status and radiographic features were the main factors distinguishing the early discontinuation group from those continuing ALIS.

Adverse events

Among the 25 patients, adverse events were observed in 72% overall, with a significantly higher incidence in the early discontinuation group (100% vs. 63%; p=0.042). Specifically, hemoptysis was the most common adverse event in early discontinuation patients (4/6, 67%). Other reasons for discontinuation included hoarseness (two patients), cough (one patient), fatigue (one patient), and fever (one patient). Multiple adverse events were reported in individual patients. In addition to these, among the six patients who discontinued early, one patient wished to discontinue due to a lack of perceived treatment effect. The remaining five patients discontinued due to specific adverse events: hemoptysis, fever, dyspnea, ALIS-related hypersensitivity pneumonitis, and diarrhea and nausea (one patient each). In contrast, the continuation group also experienced adverse events but with lower overall frequency and severity.

## Discussion

In this study, early discontinuation of ALIS was observed in 24% of patients, and we identified low BMI, hypoalbuminemia, and FC radiographic patterns as significant factors associated with this discontinuation. We believe these findings provide crucial insights for improving patient selection and management strategies in ALIS treatment for refractory MAC-PD in real-world clinical practice.

It is widely recognized that malnutrition, particularly low BMI and hypoalbuminemia, is a significant prognostic factor for worse outcomes in NTM lung disease in general [6,7,15]. Our study specifically found a strong association between poor nutritional status (low BMI and hypoalbuminemia) and early ALIS discontinuation. This might be attributed to several compounding factors: compromised systemic reserves due to malnutrition could significantly reduce patients' tolerance to adverse events associated with ALIS inhalation (e.g., cough, hoarseness, hemoptysis, dyspnea). Furthermore, the adverse events themselves might trigger a vicious cycle by causing anorexia and general malaise, thereby exacerbating pre-existing malnutrition. Additionally, impaired immune function, which hypoalbuminemia can indicate, may impede the control of existing infections, further hindering overall health improvement. Our findings provide additional perspective by highlighting the clinical relevance of poor nutritional status in relation to ALIS "tolerability" and "treatment adherence", beyond its established role as a general poor prognostic factor.

We also considered the reasons why FC lung lesions were associated with early discontinuation. The FC type generally represents a more severe form of MAC-PD, often accompanied by extensive lung destruction, severe impairment of pulmonary function, and complications such as hemoptysis [7,15]. In our cohort, hemoptysis was indeed the most common adverse event leading to discontinuation, which may be partly explained by the structural fragility of cavitary lesions and potentially exacerbated by poor nutritional status (e.g., low BMI and hypoalbuminemia) contributing to tissue vulnerability. While ALIS inhalation itself might induce local airway irritation, the underlying structural and nutritional conditions are likely to have played a predominant role. Patients with such severe conditions might be more vulnerable to ALIS-related adverse events, particularly respiratory side effects, leading to treatment discontinuation if pre-existing dyspnea or cough worsens. Moreover, extensive cavitary lesions may affect the intrapulmonary distribution and penetration of the drug, potentially leading to reduced efficacy [16] and subsequently diminishing patient motivation for treatment continuation. This suggests that for patients progressing from NB to FC type, it may be advisable to consider ALIS intervention, if possible, before the disease fully advances to the FC stage.

In our current analysis, we did not find that co-infections with *Aspergillus* or *P. aeruginosa* influenced early discontinuation. However, previous reports suggest that co-infections, such as with* P. aeruginosa*, not only worsen prognosis but also increase the severity of bronchiectasis and impair disease control [12,17-19]. It is anticipated that the frequency of co-infections will increase with longer disease duration and more severe MAC-PD, often paralleling a decline in nutritional status. From this perspective, in cases where future worsening of MAC-PD is anticipated, early treatment intensification, including ALIS, might also be effective in controlling these poor prognostic co-infections.

As expected, the overall incidence of adverse events was higher in the early discontinuation group, indicating that adverse events are indeed a major barrier to ALIS continuation. Consistent with previous reports, hemoptysis, hoarseness, cough, and fever were frequently observed [4,5,12,16]. Additionally, in one patient, a lack of perceived treatment efficacy contributed to discontinuation. For patients with the identified risk factors (low BMI, hypoalbuminemia, FC type), thorough pre-ALIS counseling regarding potential adverse events, strict post-initiation monitoring, and proactive management of symptoms, including consideration of adjusted dosing schedules (e.g., alternate-day or thrice-weekly administration), are essential for treatment adherence.

The importance of nutritional therapy has been highlighted across various respiratory diseases such as chronic obstructive pulmonary disease (COPD), interstitial lung disease, and pulmonary NTM disease [20-22]. We previously reported that early nutritional intervention is crucial in refractory pleuroparenchymal fibroelastosis (PPFE), a challenging disease like pulmonary NTM, which often presents with a high prevalence of underweight patients where emaciation itself is a poor prognostic factor [23]. This report emphasized that nutritional intervention is often too late if initiated after significant weight loss has occurred or after the lung disease has progressed to a severe stage. Instead, it stressed the importance of initiating nutritional therapy before such events occur and also provided a practical outline for nutritional intervention [24]. For instance, pulmonary function in COPD patients can be significantly improved with high-fat, high-protein, and low-carbohydrate nutritional intake [24,25]. Similar to patients with COPD, those with PPFE are often naturally underweight and usually experience progressive body weight loss after their diagnosis [23-26]. Therefore, based on nutritional support offered to COPD patients, dietitian nutritionists at our hospital usually target a body weight setting and then propose the required amount of nutritional intake with a high-fat, high-protein diet with moderate carbohydrates [23,27]. Drawing upon these insights, our institution also implements nutritional counseling for underweight patients with pulmonary NTM disease [28,29], recognizing that a proportion of these patients are known to have concomitant PPFE, which is more prevalent among lean individuals and is likely to present similar nutritional challenges. For the successful treatment of pulmonary NTM disease with multidrug antimicrobial therapy, maintaining an adequate nutritional status is a prerequisite. Therefore, the critical importance of nutritional therapy should be re-recognized.

This study has several limitations that warrant consideration. First, as a retrospective observational study, it inherently limits our ability to establish definitive causal relationships. The observed associations might be influenced by unmeasured confounding factors that were not captured in the medical records. In addition, the variables identified as associated with early discontinuation (low BMI, hypoalbuminemia, and FC type) are interrelated, and potential confounding among them cannot be excluded. Because of the small sample size, we were unable to perform multivariable analyses to adjust for such confounding, and this limitation should be kept in mind when interpreting our findings. Second, the small sample size of 25 patients may have resulted in insufficient statistical power to detect all significant differences and limits the generalizability of our findings. The results should therefore be interpreted with caution. Third, as a single-center study, the patient population and clinical practices may not be representative of other healthcare settings or broader patient demographics, which also restricts the generalizability of our conclusions. Moreover, the intervals of clinical, radiological, and microbiological follow-up were not standardized, reflecting routine clinical practice, which may have influenced the consistency of data collection and outcome assessment. Furthermore, the follow-up period was insufficient to evaluate the long-term efficacy of ALIS or the full spectrum of long-term adverse event patterns. Finally, although information on ALIS administration schedules (daily vs. alternate-day or thrice-weekly) was available, the small sample size did not allow for a detailed investigation into their relationship with tolerability and efficacy, which requires further dedicated study. Overall, the retrospective design, small sample size, and single-center setting substantially limit the generalizability of our findings, and the results should therefore be interpreted with considerable caution.

## Conclusions

This study identified significant clinical and nutritional risk factors associated with the early discontinuation of ALIS in patients with refractory MAC-PD, specifically low BMI, hypoalbuminemia, and FC radiographic patterns. These findings highlight the importance of early intervention and close monitoring of patients. When considering ALIS administration, clinicians should be mindful of the patient's nutritional status before significant weight loss occurs and before the development of FC-type disease. For patients presenting with these identified risk factors, a meticulous assessment prior to ALIS initiation and rigorous post-initiation management focusing on nutritional support and stringent monitoring for adverse events may contribute to improved treatment adherence and clinical outcomes.
